# Mitofusin Activation Delays Aging in *Caenorhabditis elegans*

**DOI:** 10.3390/cells15191789

**Published:** 2026-09-30

**Authors:** Jochen Weigele, Lihong Zhang, Jiajia Li, Elisabeth Roider, Caroline E. Walton, Gerald W. Dorn

**Affiliations:** 1 Department of Regenerative Medicine and Cell Biology, Medical University of South Carolina, 171 Ashley Ave, Charleston, SC 29425, USA; weigelej@musc.edu; 2 Department of Internal Medicine, Center for Pharmacogenomics, Washington University in St. Louis, Saint Louis, MO 63110, USA; lzhang25@wustl.edu (L.Z.); lijiajia@cpl.ac.cn (J.L.); walton.caroline@mayo.edu (C.E.W.); 3 Mitochondria in Motion, Inc., Saint Louis, MO 63110, USA; 4 Department of Dermatology, University Hospital of Basel, 4031 Basel, Switzerland; elisabeth.roider@usb.ch

**Keywords:** mitochondrial fusion, lifespan, healthspan

## Abstract

**Highlights:**

**What are the main findings?**

- Mitofusin activators prolonged survival and enhanced the physical fitness of *C. elegans*.
- Mitofusin activation increased *C. elegans* resistance to thermal and oxidative stress.

**What is the implication of the main findings?**

- Age-related mitochondrial degeneration and its downstream consequences can be mitigated in worms by pharmacological activation of fusion-mediated mitochondrial repair.

**Abstract:**

Age-linked tissue dysfunction is associated with mitochondrial degeneration manifested as organelle fragmentation and respiratory dysfunction. Efforts to mitigate downstream consequences of mitochondrial degeneration, as with ROS scavengers, have not translated to the human condition. We posit that intrinsic fusion-mediated mechanisms for mitochondrial protection modulate aspects of aging linked to mitochondrial abnormalities. In *C. elegans* roundworms mitochondrial fusion is mediated by the mitofusin ortholog Fuzzy Onion-1 (fzo-1). Here, the addition of a mitofusin inhibitor, MFI8, to cultures of age-synchronized *C. elegans* shortened worm lifespan in a concentration-dependent manner. Reciprocally, the mitofusin activators Chimera and 8015-P2 prolonged worm survival in a dose-dependent manner. The pro-survival effect of Chimera was absent in worms lacking its protein target, FZO-1, and at lower concentrations Chimera-amplified survival was further increased by absence of the mitochondrial fission protein that acts in opposition to fzo-1, DRP-1. The mitofusin activator 8015-P2, which is more potent than Chimera, exhibited greater anti-aging effects on lifespan and improved worm healthspan measured as forced swimming activity and resistance to thermal and oxidative stresses. These proof-of-concept worm studies identify aging phenotypes reciprocally related to fzo-1/mitofusin activation status. As 8015-P2 is under development for use in human neurodegenerative diseases, it might alleviate aging phenotypes evoked by mitochondrial degeneration in higher species.

## 1. Introduction

Aging in most multicellular animals is associated with cellular deterioration/senescence, a progressive decline in physiological organ functioning, and ultimately death. Accordingly, death might be delayed by prolonging the normal performance of critical organ systems by interrupting pathways leading to cellular senescence. The observation that aging phenotypes develop and progress at a variable rate among different persons is evidence that concomitant chronic disease increases the risk for premature or accelerated aging [1,2,3]. Mitochondrial dysfunction has long been recognized as a common underlying feature of etiologically diverse age-associated chronic diseases, consistent with the ideas that mitochondrial health is essential to normal cell and organ functioning and that it is impaired during aging [4].

Mitochondria are ubiquitous intracellular organelles that produce chemical energy in the form of adenosine triphosphate (ATP), which fuels normal cell functioning and, depending on the pathophysiological context, cell repair, removal and regeneration. Mitochondrial damage or dysfunction can uncouple respiration from ATP production, thereby reducing cellular energy stores while increasing elaboration of cytotoxic reactive oxygen species (ROS) [5]. For this reason, many investigational attempts to optimize mitochondrial health during aging have focused on improving mitochondrial metabolism or reducing mitochondrial-derived ROS [6,7,8]. The results of basic studies of this nature have generally supported the concept that mitochondrial fitness is an important determinant of cellular senescence and aging, but translating these findings to the clinic has been challenging.

For the past several years our and other research groups have evaluated a different approach that targets mitochondrial health in some age-related diseases by genetically overexpressing mitofusins (Mfns) [9] or by pharmacologically enhancing Mfn activity [10,11] in order to enhance fusion-mediated mitochondrial repair. The results of such studies support the notion that mitochondrial fitness and resistance to injury can be improved by enhancing mitochondrial fusion mediated in part by Mfn1 and Mfn2. Fusion-mediated content exchange is a principal mechanism for mitochondrial rejuvenation, promoting organelle repair by mutual complementation of mitochondrial DNA and by dilution of damaged mitochondrial lipids and proteins [12]. Consistent with this paradigm, mitofusin activation has delayed or reversed some pre-clinical models of age-associated neurodegenerative diseases [13,14,15,16]. Reciprocally, genetic ablation of mitofusins or mutational disruption of mitofusin function has promoted cell senescence and evoked premature aging phenotypes in skeletal and heart muscles and neuronal tissue [16,17,18,19,20,21,22]. What is not known is whether mitofusin activation might have palliative effects on normal aging.

Here, we tested the hypothesis that enhancing mitochondrial fusion through stimulation of mitofusin activity can delay aging phenotypes and prolong life. To do so we took advantage of existing genetic models that perturb mitochondrial fusion and fission in the nematode *C. elegans*, which has a highly reproducible and predictable life cycle that lends itself to aging studies [23]. Our results extend the idea that mitochondrial health is linked to mitochondrial fusion in normal aging and show for the first time that pharmacological mitofusin activation can not only extend longevity (quantity of life) but also markedly improve organism fitness (quality of life) during the aging process.

## 2. Materials and Methods

### 2.1. C. elegans Strains and Maintenance

The *C. elegans* strains used in this study were N2 Bristol (wild-type), BXN723 [fzo-1(cjn20) II.], CU6372 [drp-1(tm1108) IV.] and SD1347 [ccIs4251 I; stIs10079.], which were provided by the Caenorhabditis Genetics Center (CGC) at the University of Minnesota, Minneapolis MN, USA. Worm strains were maintained long-term at 18 °C in nematode growth medium (NGM) plates seeded with *Escherichia coli* strain OP50 obtained from Carolina Biological Supply (Burlington, NC, USA) [24].

### 2.2. Mitofusin-Modulating Compounds

The mitofusin activators Chimera [25] and 8015-P2 [16] were synthesized (>95% purity) at WuXi Apptech Co., Ltd. (Shanghai, China) and provided by Mitochondria in Motion Discovery, Inc. (Sullivan’s Island, SC, USA) under terms of a Material Transfer Agreement. The mitofusin inhibitor MFI8 [26] was purchased from Ascendex Scientific (Levittown, PA, USA; Cat.# AS5434). Stock solutions (10 mM) of all compounds were prepared in DMSO and maintained at −20 °C until use.

### 2.3. Mitochondrial Studies in Cultured Fibroblasts

The change in mitochondrial aspect ratio (length/width) provoked by MFI8 was assessed in wild-type C57/bl6 mouse embryonic fibroblasts (MEFs) cultured overnight (~14 h) in the presence of the inhibitor at a final concentration range of 0.5 nM–10 μM (diluted 1:1000 from DMSO stocks) or the DMSO vehicle. Cells were stained with MitoTracker Orange (200 nM for 10 min). Confocal imaging of live cells was performed on a Keyence BZ-X800 fluorescence microscope (Osaka, Japan) using the 40x objective; three independent experiments were performed. The average mitochondrial aspect ratio for at least 10 cells per condition per experiment was calculated using ImageJ (version 1.54p) by the National Institutes of Health. MFI8 effects to reduce the mitochondrial aspect ratio, i.e., to induce mitochondrial fragmentation, were compared to those of the fusion-inhibitory peptide (MFN2 398–418 [MP2]; 5 mM) [27]. Concentration-response curves were generated using a sigmoidal model in Prism 10 software (version 3.0). The IC_50_ value and variance were calculated from interpolated data of all replicate experiments.

### 2.4. Age Synchronization and Worm Longevity Experiments

Age-synchronized nematodes were obtained by transferring 10 adult worms to a 60mm NGM agar petri dish seeded with *E*. *coli* strain OP50 [24,28], removing the adult worms after 3 h of egg laying, and incubating the plates at 22 °C. Upon reaching the L4 stage at least 20 worms were transferred to a 6-well plate containing NGM agar seeded with *E*. *coli* OP50 plus 40 μM 5-fluoro-2′-deoxyuridine (FUdR, Sigma-Aldrich, Burlington, MA, USA; Cat.# F0503) to prevent progeny growth [29]. Mitofusin activity modulators were included in the feeding layer at a concentration of 1 µM, 10 µM, or 100µM, and DMSO was used as the vehicle control. Worm cultures for longevity studies were maintained at 22 °C and live and dead animals were counted daily. Animals that were not active when gently prodded using a worm pick were scored as dead. Worms were transferred to fresh NGM plates with mitofusin-modulating compounds twice every week. The “bump” in worm survival observed in several experiments here and previously [30] is likely due to this re-feeding step. A few worms crawled off the NGM plates and were excluded from analyses.

### 2.5. In Situ Studies of C. elegans Mitochondria

*C. elegans* strain SD1347 expresses both a mitochondria-targeted GFP transgene and a nuclear-targeted GFP transgene in body-wall muscle cells [31]. Worms of this strain at different ages were transferred to glass slides in 100 mL of M9 buffer (85.6 mM NaCl, 42.4 mM Na_2_HPO_4_, 22 mM KH_2_PO_4_, 1 mM MgSO_4_) supplemented with 5 mM levamisole HCl for immobilization. Confocal images were obtained on a Nikon Eclipse Ti2 confocal microscope using the Nikon CGI Plan Apo VC 60x A/1.20 WI water immersion objective (Nikon Corporation, Tokyo, Japan) [32]. The mitochondrial length of at least 10 animals per condition (3 images per animal) was calculated using NIH Image J (version 1.54p).

### 2.6. Swimming (Body Bending) Assay

The rate and extent of body bending (thrashing) after immersion in phosphate-buffered saline (PBS) were assayed using the WormLab Imaging System’s ^®^ automated multiworm tracker function (version 2024.01.01) (MBF BioScience, Williston, VT, USA). For each condition, at least 10 animals were transferred to a 35 mm culture dish and 1ml of PBS was added. Thirty seconds after immersion a 2 min video was captured (10 frames/second) and the number and magnitude of body bends (thrashes) per minute were automatically quantified. Data are reported as thrashes/minute after immersion.

### 2.7. Thermal Stress Assay

For each experiment, approximately 20 age-synchronized DOA 5 worms cultured from DOA 1 with the mitofusin activator 8015-P2 (100 mM) or vehicle were transferred to fresh NGM agar plates containing 2.5% agar (without *E. coli*). After equilibration for 15 min at room temperature the worm cultures were transferred to a 37 °C incubator and the number of living worms was assessed (vide supra) after 4, 6 and 8 h of thermal stress.

### 2.8. Oxidative Stress Survival Assay

Age-synchronized L4-stage N2 Bristol *C. elegans* specimens were cultured for four days in the presence of 8015-P2 (100 mM) or the vehicle. DOA 5 worms were washed off plates with 1ml of M9 buffer and centrifuged at 400× *g* for 2 min, and the worm pellet was washed twice with 1ml of M9 buffer. The *C. elegans* response to H_2_O_2_ is complex: sub-lethal concentrations (10–100 μM) trigger avoidance and feeding inhibition, while 1–2 mM is acutely toxic within 24 h and is the standard concentration range for oxidant stress assays [33,34]. Based on preliminary studies using 1 and 2 mM we chose 2 mM as the concentration most suitable to show a protective effect. At least 20 worms per experiment were incubated with 2mM hydrogen peroxide in M9 buffer at room temperature and the number of living animals was assessed after 2, 3 and 4 h. Worms that were not active when gently prodded using a worm pick were scored as dead.

### 2.9. Measurement of Intracellular ROS

Age-synchronized L4-stage *C. elegans* specimens were cultured in the presence of 8015-P2 (100 mM) or the vehicle. DOA 4 worms were washed with 1 mL of M9 buffer and centrifuged at 400× *g* for 2 min, and the worm pellet was washed twice with 1 mL of M9 buffer. Worms were incubated in 1 mL of 5 μM 2′,7′-dichlorodihydrofluorescein diacetate (Thermofisher, Waltham, MA, USA; Cat.# D399) in M9 buffer for 1 h in the dark and washed three times with M9 buffer. At least 20 nematodes per condition were transferred to 24-well Cellvis (Sunnyvale, CA, USA) glass-bottom plates (Cat# P14-1.5H-N) containing 1 mL each of M9 buffer + 5 mM levamisole, and they were imaged on a Keyence (Osaka, Japan) BZ-X800 fluorescent microscope (excitation: 470 nm/emission: 525 nm). The fluorescence intensity of whole worms was quantified using NIH ImageJ software (version 1.54p). The average fluorescence (arbitrary units) of all vehicle-treated animals was considered to be 100% and the fluorescence of each 8015-P2-treated worm was normalized to that value. Results are reported as % vehicle-treated.

### 2.10. Statistical Analysis

Alignment of *C. elegans* fzo-1 (UniProt ID: Q23424) and human MFN2 (UniProt ID: O95140) amino acid sequences was performed in the T-COFFEE Multiple Sequence Alignment Server (https://tcoffee.crg.eu/apps/tcoffee/do:regular) accessed on 14 July 2026 [35].

Unless otherwise stated all experiments were independently repeated at least three times and results are presented as box-and-whisker plots or line graphs with means ± SEMs. Statistical significance for lifespan differences was analyzed by the log-rank test using Prism 10 (version 3.0). Paired comparisons used unpaired Student’s *t*-tests; multi-group comparisons used one- or two-way ANOVA as specified, along with Tukey’s post hoc comparison using Prism 10 (version 3.0). *p* < 0.05 was considered significant.

## 3. Results

### 3.1. Mitofusin Inhibition with MFI8 Decreases Longevity in C. elegans

**Figure 1:**
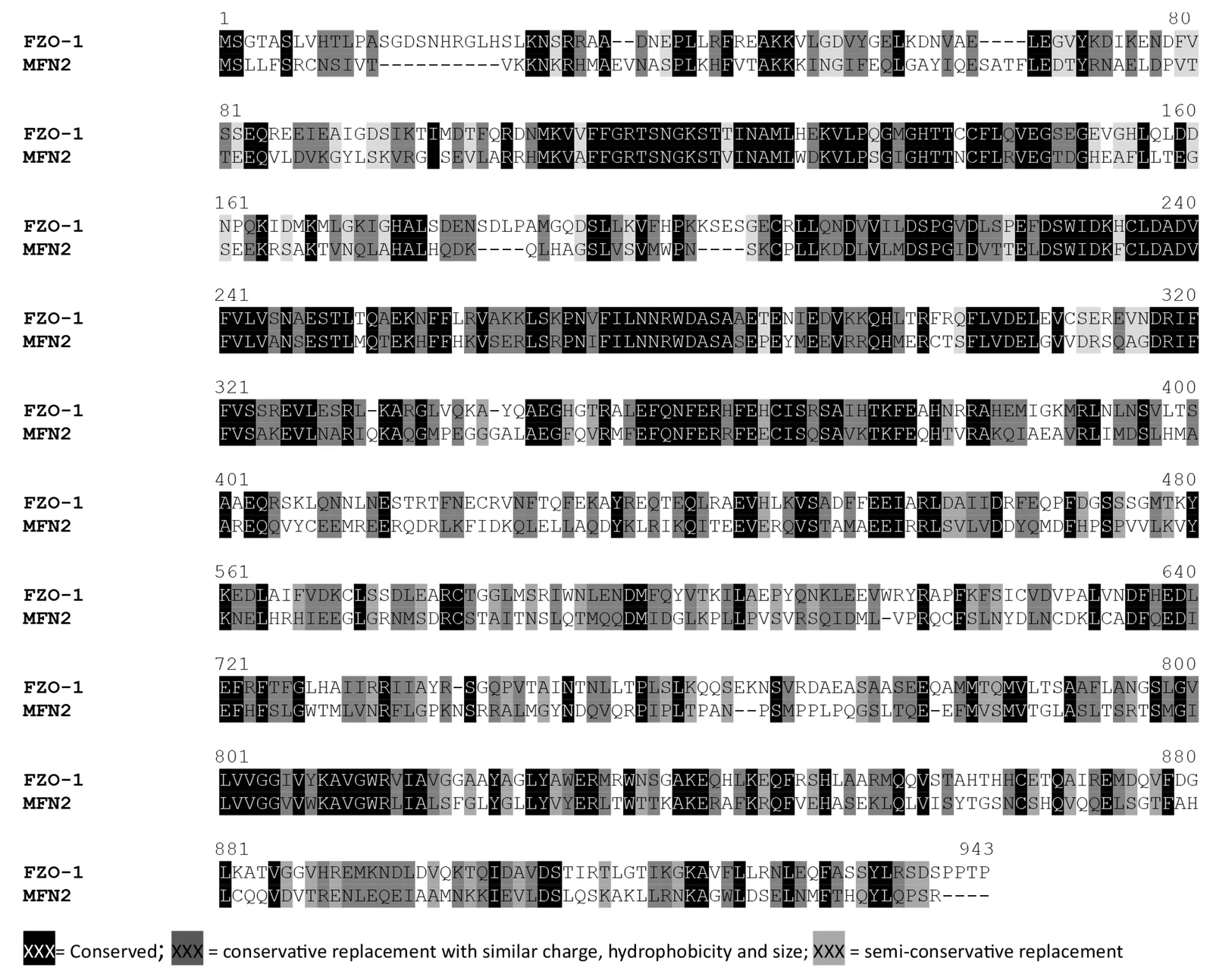
Amino acid similarity between *C. elegans* FZO-1 and human MFN2.

The ability to specifically manipulate mitochondrial fusion using pharmacology opens new doors for translational investigation. *C. elegans* has only a single “mitofusin” protein, which is designated FZO-1 (fuzzy onion) [36]. Despite modest amino acid sequence identity (36%), FZO-1 and MFN2 have extensive amino acid conservation with similar predicted structures and biological functions (Figure 1). Additionally, like mitofusins in mammals, FZO-1 is essential for normal worm life- and healthspan [37,38,39,40].

**Figure 2:**
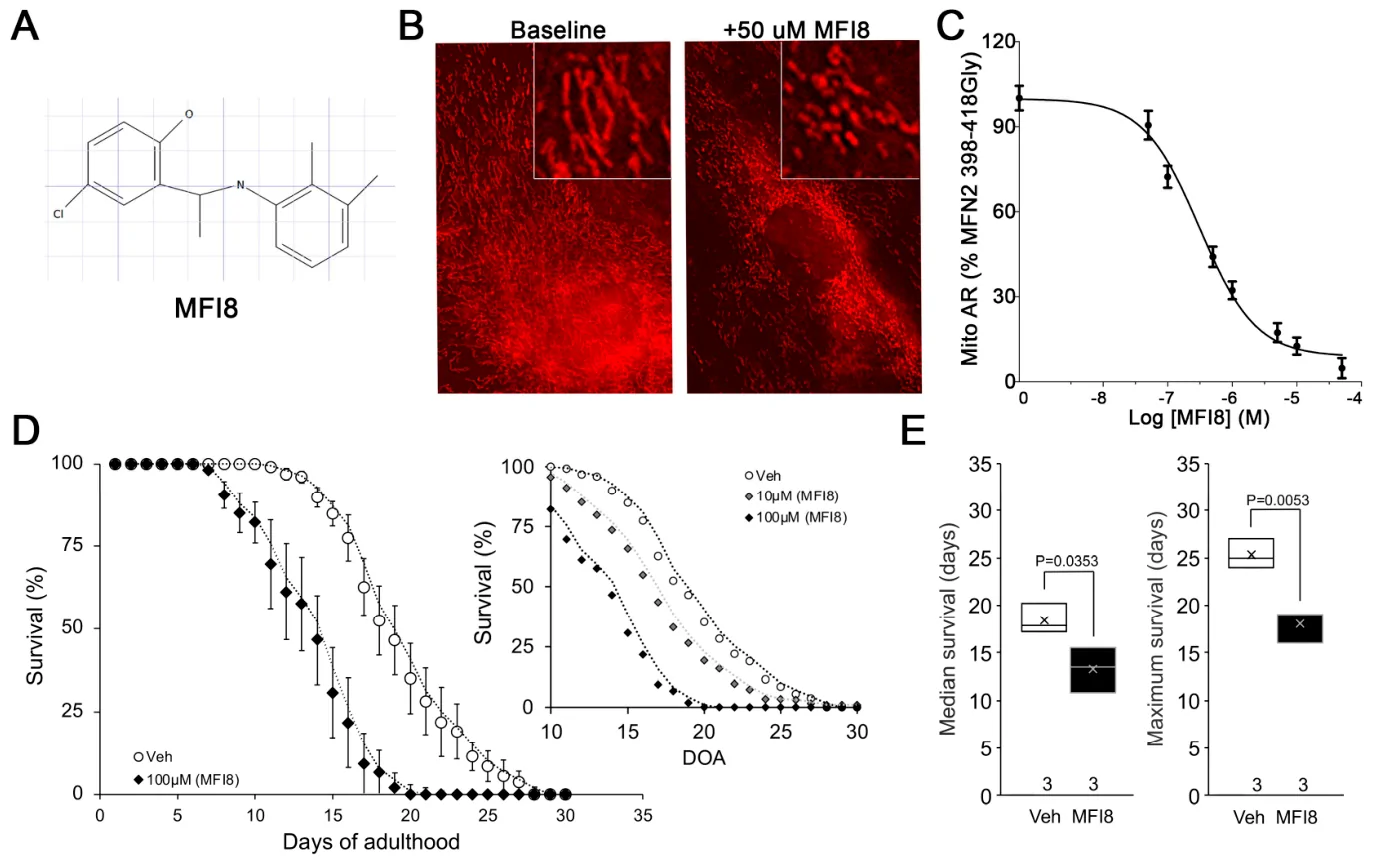
Mitofusin inhibition with MFI8 reduces C. elegans longevity. (**A**). Chemical structure of MFI8. (**B**). Representative confocal images of murine embryonic fibroblasts (MEFs) showing MFI8-induced mitochondrial fragmentation and reductions in mitochondrial aspect ratio (AR). (**C**). Group quantitative data (*n* = 3) showing the dose-dependent reduction in MEF mitochondrial AR by MFI8, reflecting inhibition of mitochondrial fusion. (**D**). *C. elegans* survival when exposed to the vehicle (open circles) or MFI8 (100 μM, black diamonds). Each point is the mean ± SEM of 3 independent biological replicates. The *inset* (expanded view—days 10–30) shows the mean values for different doses. (**E**). Quantitative group analysis of median (**left**) and maximal (**right**) survival in studies shown in (**D**). Here and below, bars encompass the 1st–3rd interquartile range, the internal horizontal line is the median value, the internal X is the mean value, and whiskers show the range of values. *N* values represent independent experiments performed with at least 20 worms per condition; *p* values vs. the WT are the result of the unpaired 2-tailed *t*-test.

The commercially available compound 4-chloro-2-(1-((2,3-dimethylphenyl)amino) ethyl)phenol, designated “MFI8” by Zacharioudakis et al. [26] (Figure 2A), reportedly inhibits mitofusins by impeding the essential transition from a closed conformation to an open conformation [25,41]. We confirmed that MFI8 suppresses mitochondrial fusion in a dose-dependent manner using normal murine embryonic fibroblasts (MEFs); the IC_50_ was 3.1 μM (95% CI 2.19–4.40 μM) (Figure 2B,C), which is similar to the previously reported IC_50_ of 4.8 μM [26]. Importantly, we used the same reference mitofusin inhibitor (a MFN2-derived antagonist peptide called MFN2 398-418Gly) [27] as the previous MFI8 study. Thus, MFI8 is a validated mitofusin inhibitor.

Genetic ablation of Mfn/FZO-1 in *C. elegans* reportedly decreases the median (but not maximal) lifespan [37]. We asked if mitofusin inhibition with MFI8 would recapitulate this phenotype. Because MFI8 bioavailability (the fraction of the administered compound that is delivered to the target) in *C. elegans* and its effect on the worm FZO-1 mitofusin homolog are unknown, our proof-of-concept studies using a high concentration (100 μM) of the inhibitor or vehicle (1% DMSO) added to the feeding soft agar layer of synchronized cultures of newly molted adults; the number of live worms was determined daily thereafter. At this concentration, MFI8 adversely impacted worm survival (Log-rank Chi square = 72.76; *p* < 0.0001) (Figure 2D). MFI8 reduced by ~40% both median survival (from 18.4 ± 0.9 to 13.2 ± 1.4 days) and maximal lifespan (from 25.3 ± 0.9 to 18 ± 1 days) (Figure 2E). At a concentration closer to its in vitro IC_50_ for mammalian mitofusins (10 μM) the negative survival effect of MFI8 was approximately halved (see Figure 2D, inset). Thus, mitofusin inhibition has dose-dependent adverse effects on worm longevity.

### 3.2. Mitofusin Activation with Chimera Increases Longevity in C. elegans

**Figure 3:**
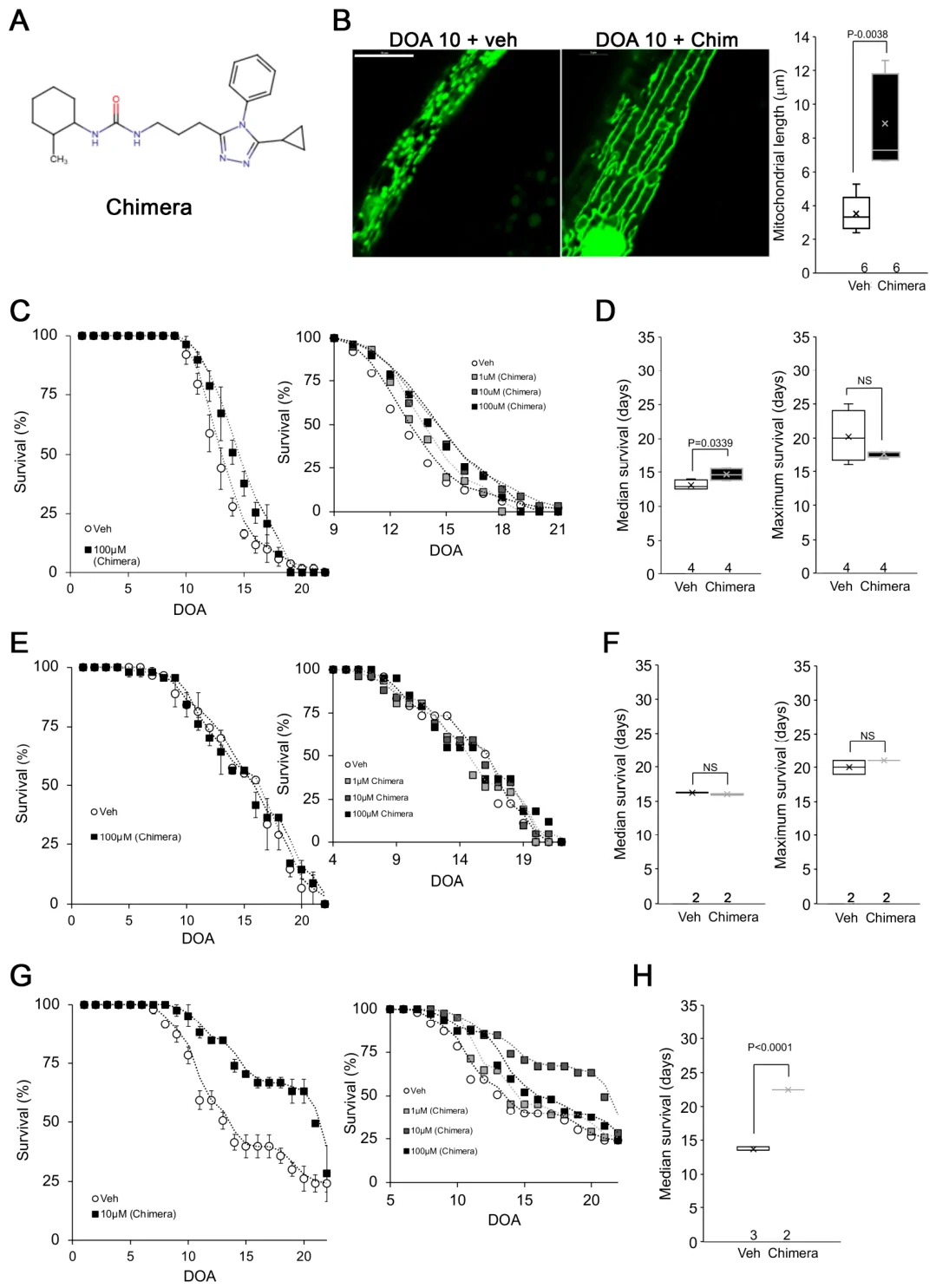
Mitofusin activation with Chimera increases C. elegans longevity. (**A**). Chemical structure of Chimera. (**B**). Representative confocal images of live *C. elegans* (day 10) with transgenic mitochondrial GFP showing Chimera-induced mitochondrial elongation (right panel) compared to vehicle-exposed worms (left panel). (**C**). *C. elegans* survival when exposed to the vehicle (open circles) or Chimera (10 μM, black squares). Each point is the mean ± SEM of 4 independent biological replicates. The *inset* (expanded view—days 9–21) shows mean values for 1, 10, and 100 μM Chimera. (**D**). Quantitative group analysis of median (**left**) and maximal (**right**) survival in studies shown in (**C**). (**E**,**F**). Same experiment as in (**C**,**D**) but performed in *C. elegans* mutants lacking the worm mitofusin, FZO-1. (**G**,**H**). Same experiment as in (**C**,**D**) but performed in *C. elegans* mutants lacking the worm mitochondrial fission protein DRP-1. *N* values represent independent studies of at least 20 worms per condition; *p* values vs. the WT are the result of the unpaired 2-tailed *t*-test; NS = non-significant (*p* > 0.05).

The prototype small-molecule mitofusin activator is N-(trans-4-hydroxycyclohexyl)-2-(3-phenylpropyl)-trans-(R,R) cyclopropane-1-carboxamide, designated “Chimera” (Figure 3A) [25], which has been the principal comparator for subsequent, chemically diverse mitofusin activators [13,42,43]. Chimera’s ability to reverse mitochondrial abnormalities by allosterically promoting mitochondrial fusion is well established in multiple in vitro models of human neurodegenerative diseases [13,15,16,25,42,43,44,45]. Accordingly, our initial studies of mitofusin activation in *C. elegans* aging employed Chimera.

Mitochondrial fragmentation (shortening of mitochondria) is a typical feature of senescence and is observed in both mammalian and roundworm aging (vide infra) [46,47]. When Chimera (100 μM) was added to the feeding layer of synchronized cultures on the first day of adulthood (DOA), the mitochondria of DOA 10 *C. elegans* worms expressing mitochondria-targeted green fluorescence protein (GFP) were elongated compared to vehicle (1% DMSO)-treated controls (Figure 3B). This more youthful mitochondrial phenotype was associated with a modest increase in longevity (Log-rank Chi square = 10.67; *p* < 0.0011) (Figure 3C). Chimera increased the median survival time by 12%, from 13.0 ± 0.36 days in vehicle-treated worms to 14.65 ± 0.46 days with Chimera treatment (Figure 3D, left); maximal survival time was not changed (Figure 3D, right). Extended survival in Chimera-treated worms appeared to be dose-dependent (Figure 3C, *inset*).

FZO-1-mutant *C. elegans* specimens lack endogenous worm mitofusin. Consequently, there were no life-prolonging effects of Chimera at any dose in FZO-1-mutant *C. elegans* (Figure 3E,F). This result mechanistically links the pro-survival effect of Chimera to its activation of mitofusins and promotion of mitochondrial fusion.

Dynamin-related protein 1 (DRP-1) mediates mitochondrial fission, acting in opposition to FZO-1/MFN. We asked if the pro-survival effects of DRP-1 deletion and FZO-1 activation with Chimera might be additive, i.e., if survival could be prolonged even further in worms wherein mitochondrial fission was interrupted concomitant with enhancement of mitochondrial fusion. Remarkably, lower concentrations of Chimera provided additional survival benefits in DRP-1-mutant worms (Figure 3G,H), whereas the highest concentration (100 μM) was less effective (Figure 3G, inset). We interpret this finding as revealing a therapeutic limit to altering the balance of mitochondrial dynamism in favor of fusion.

### 3.3. Mitofusin Activator 8015-P2 Extends C. elegans Survival

**Figure 4:**
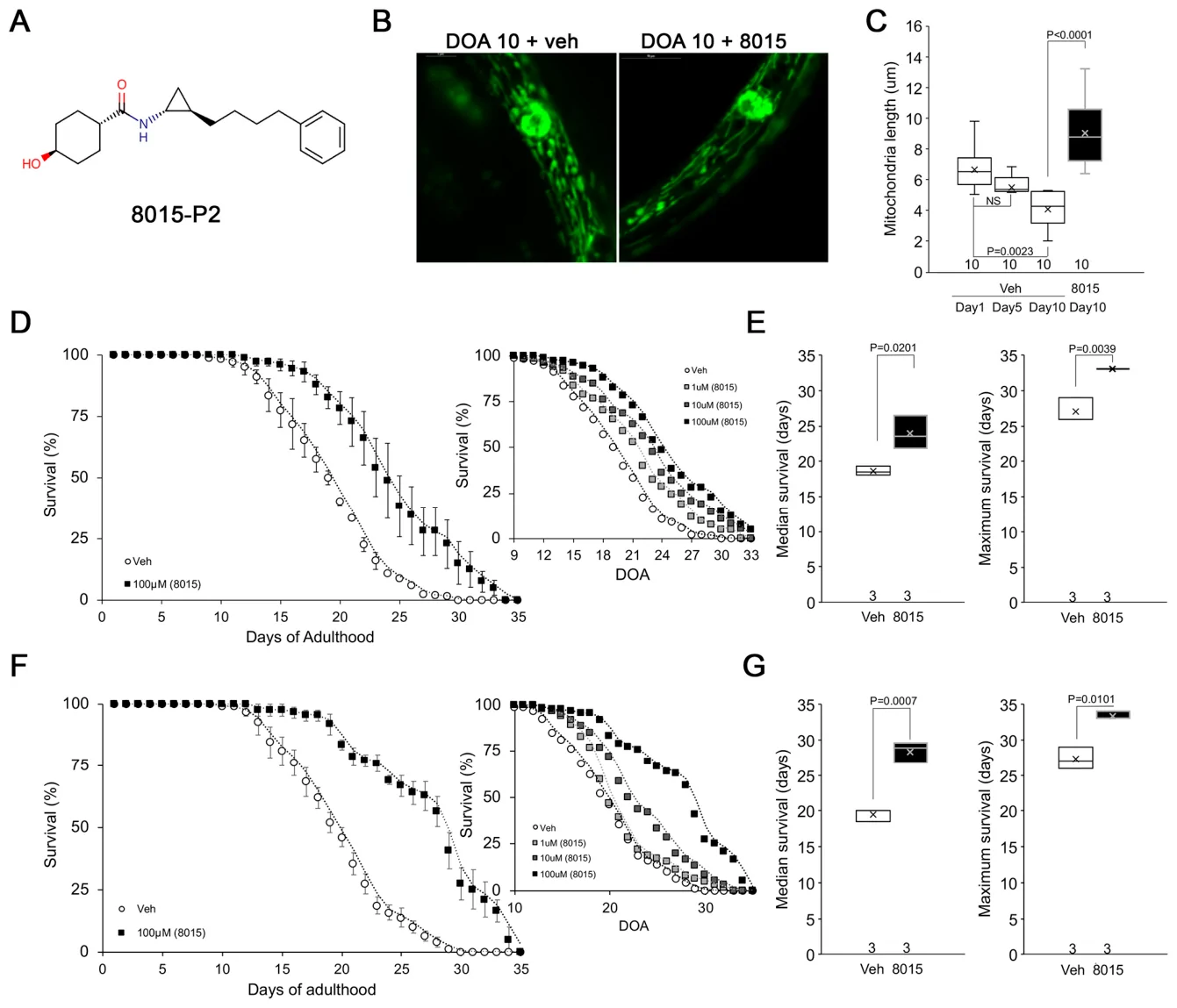
Mitofusin activator 8015-P2 prolongs C. elegans survival when administered throughout adulthood or from day 5. (**A**). Chemical structure of 8015-P2. (**B**). Representative confocal images of live mito-GFP-expressing *C. elegans* (DOA 10) showing 8015-P2-induced mitochondrial elongation (**right panel**) compared to vehicle-exposed worms (**left panel**). (**C**). Mitochondrial length in vehicle-treated *C. elegans* (white boxes) at DOAs 1, 5, and 10 and DOA 10 8015-P2 treated worms (black box) (**D**). *C. elegans* survival when cultured with the vehicle (open circles) or 8015-P2 (100 μM, black squares) from DOA 1. Each point is the mean ± SEM of 3 independent biological replicates. The *inset* (expanded view—days 9–33) shows mean values for 1, 10, and 100 μM 8015-P2. (**E**). Quantitative group analysis of median (**left**) and maximal (**right**) survival in studies shown in (**D**). (**F**,**G**). Same experiment as in (**D**,**E**) with treatment started at DOA 5. *N* values in (**C**) represent individual worms, and those elsewhere are individual experiments of at least 20 worms per condition; *p* values in (**C**) used one-way ANOVA with Tukey’s test, and in (**E**,**G**) we used an unpaired 2-tailed *t*-test; NS = non-significant (*p* > 0.05).

Chimera is a useful mitofusin activator in vitro, but it is rapidly eliminated by first-pass hepatic metabolism in mice [44]. This characteristic fatally limits Chimera’s clinical application. By comparison, (1r,4r)-4-hydroxy-N-[(1R,2R)-2-(4-phenylbutyl)cyclopropyl]cyclohexane-1-carboxamide, designated 8015-P2 (Figure 4A), is a third-generation allosteric mitofusin activator with favorable in vivo pharmacokinetics and an ability to reverse mitochondrial dysfunction in genetically modified mice [16]. Because this compound has the potential to advance to human trials, and to validate the pro-survival effect of mitofusin activators using chemically dissimilar compounds, we examined 8015-P2 effects on *C. elegans*.

First, we measured muscle mitochondrial morphology in worm wall muscle cells as a function of age and 8015-P2 treatment status. Using the same technique as in Figure 3B, we observed mitochondrial fragmentation (a reduction in length) in vehicle-treated worms between DOAs 5 and 10 (Figure 4B,C, white boxes). The addition of 8015-P2 (100 μM) at DOA1 prevented this age-associated decrease in mitochondrial length (Figure 4B,C, black box).

As with Chimera, the addition of 8015-P2 (100 μM) to the feeding layer of DOA1 worms extended *C. elegans* lifespan, improving overall longevity (Log-rank Chi square = 35.09; *p* < 0.0001) (Figure 4D), increasing median survival by 28% from 18.6 ± 0.4 to 23.9 ± 1.4 days, and prolonging maximal lifespan by 22% (from 27 to 33 days) (Figure 4E). Compared to the same concentration of Chimera, 8015-P2 was more efficacious, increasing median survival approximately two-fold greater and prolonging maximal lifespan (compare Figure 3D to Figure 4E). Increased efficacy of 8015-P2 versus Chimera likely reflects its ~10-fold greater fusogenic potency (~0.5 nM vs. ~5 nM measured in mouse cells) [16,25]. As expected, the life-prolonging effects of 8015-P2 were dose -dependent (Figure 4D, *inset*).

In each of the studies described above worms were exposed to mitofusin-modulating agents beginning on DOA 1. We posited that pro-survival effects would diminish if mitofusin activation was initiated later in life (but before the worms started dying on DOAs 10–11). We tested this notion by performing a survival study in which 8015-P2 was added beginning on DOA 5. Remarkably, delayed exposure to the mitofusin activator provided the same survival benefit as (adult) life-long treatment (compare Figure 4D,F). At the 100 μM concentration, 8015-P2 initiated on DOA 5 improved longevity (Log-rank Chi square = 71.95; *p* < 0.0001), increasing median survival by 46% (from 19.5 ± 0.5 to 28.4 ± 0.8 days; *p* = 0.0007) and extended maximal lifespan by 17% (from 27.3 ± 0.9 to 32 ± 1 days; *p* = 0.0031) (Figure 4G). Again, these effects were dose-dependent (Figure 4F, *inset*). Thus, mitofusin activation with compound 8015-P2 prolonged *C. elegans* survival either when provided from the beginning of adult life or in mature worms.

### 3.4. Mitofusin Activator 8015-P2 Promotes a Youthful Phenotype in Aging C. elegans

**Figure 5:**
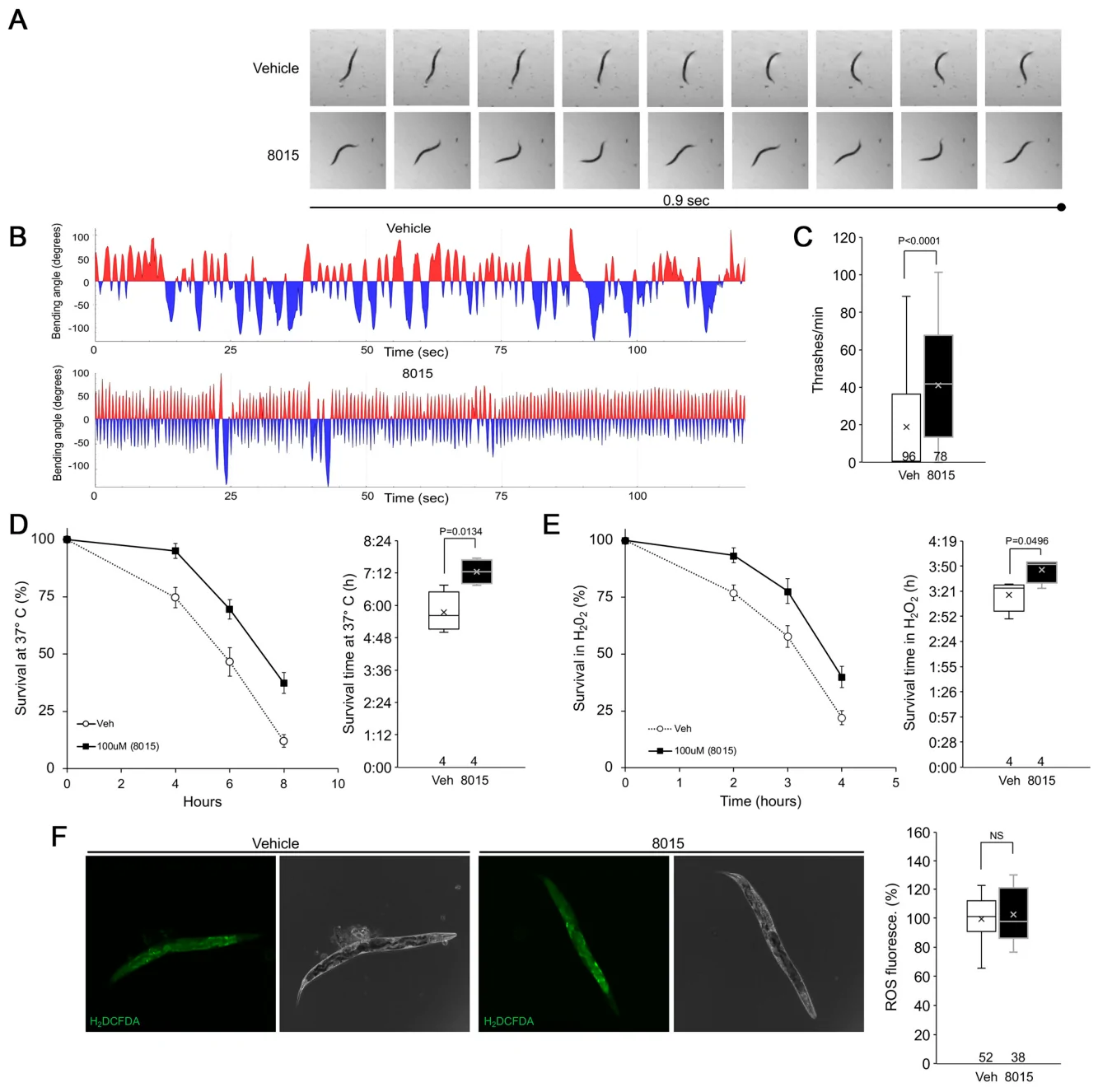
Mitofusin activation confers a youthful phenotype in *C. elegans*. (**A**). Sequential video frames (10/s) showing different thrashing responses of DOA 10 *C. elegans* after immersion in the buffer. Here and below worms were exposed from DOA 1 to the vehicle or 8015-P2 (100 μM). (**B**). Graphic data for the complete swimming studies of worms in (**A**) as reported by WormLab. (**C**). Quantitative group worm swimming data. (**D**,**E**). *C. elegans* survival under acute severe thermal (**D**) or oxidative (**E**) stress. **Left panels** are time x survival curves (statistics are reported in the text); **right panels** compare median survival times. *N* = 4 independent studies of at least 20 worms each. (**F**). Measurement of in situ ROS levels in DOA 10 *C. elegans*. Representative fluorescent (green) and optical (gray) images are shown for the vehicle- (**left pair**) and 8015-P2-treated (**right**) worm. Quantitative data are to the right. *N* values reported in (**C**,**F**) represent individual worms; *p* values in the box-and-whisker graphs used the unpaired 2-tailed *t*-test; NS = non-significant (*p* > 0.05).

Survival time (quantity of life) is of limited value in the absence of improved overall functional capacity (quality of life). *C. elegans* quality of life, or healthspan, is evidenced by locomotor activity that declines linearly with age [48]. We assessed quality of life as neuromuscular activity in *C. elegans* stressed by immersion in a buffer (forced swimming) [49]. Swimming was compared in DOA 10 worms cultured from DOA 1 without (i.e., with vehicle only) or with (100 μM) 8015-P2. Worm responses to liquid immersion were automatically imaged and quantified without bias using WormLab ^®^ [50]. As shown in representative video sequence frames, DOA 10 worms cultured with 8015-P2 reacted more vigorously to saline immersion, with greater extent and frequency of body bending than vehicle-treated worms studied in parallel (Figure 5A,B). Quantitative group analysis showed that mitofusin activation with 8015-P2 approximately doubled the average vigor of the swimming response measured as thrashes/minute (Figure 5C).

Another metric of fitness or healthspan in *C. elegans* is resistance to thermal or oxidative stress, which is maximal between DOA1 and DOA5 and declines thereafter [51]. We assayed worm survival after raising the ambient temperature from 21 °C to 37 °C. As shown in Figure 5D (left panel), DOA 10 worms exposed to 8015-P2 from DOA 1 survived longer at the higher temperature than those cultured in the vehicle alone (2-way ANOVA: veh vs. 8015, *p* = 1.2 × 10^−6^; time factor *p* = 8.9 × 10^−16^; interaction between treatment groups and time *p* = 0.0099). A different metric, median survival time at 37 °C, was likewise increased by the mitofusin activator (Figure 5D, right panel).

A similar experiment interrogated *C. elegans* sensitivity to oxidative stress by exposing DOA 10 vehicle- or 8015-P2-treated (from DOA 1) worms to H_2_O_2_ (2 mM). Worms treated with the mitofusin activator were less sensitive to H_2_O_2_ than those cultured in the vehicle alone (Figure 5E, left panel) (2-way ANOVA: veh vs. 8015, *p* = 2.33 × 10^−5^; time factor *p* = 4.77 × 10^−15^; interaction between treatment groups and time *p* = 0.0451). The median survival time during oxidative stress was modestly increased by the mitofusin activator (Figure 5E, right panel). Finally, we asked if increased resistance to oxidative stress in worms cultured with the mitofusin activator might be attributable to reduced endogenous ROS elaboration and used 2′, 7′-dichlorofluorescein diacetate (H_2_DCFDA) [52,53] to measure ROS levels in worms cultured in the vehicle or 8015-P2 from DOA 1. Mitofusin activation with 8015-P2 did not have any measurable effect on in situ ROS levels at DOA 10 (Figure 5F).

## 4. Discussion

These studies demonstrate that pharmacological mitofusin activation extends lifespan and enhances healthspan in *C. elegans*. Aging is a natural phenomenon that will affect all members of a population fortunate enough to survive long enough. Although aging is inevitable, environmental and genetic factors can impact the progression and extent of age-related phenotypes. For example, diseases that mimic the natural time-dependent deterioration in organ dysfunction can spread across organ systems and accelerate aging [54]. Acting in opposition to disease, aging can be mitigated with exercise or dietary restriction, which are thought to positively impact mitochondria [46,55]. Indeed, while precise causes of aging and disease-mediated premature senescence are incompletely defined, a common factor seems to be mitochondrial dysfunction. For this reason, we and others are targeting mitochondria in aging. A common approach has been to attack the downstream consequences of mitochondrial damage, as with ROS scavenging [56]. By contrast, mitofusin activation works upstream, acting directly upon mitochondria to promote resistance to and recovery from organelle injury.

The small roundworm *C. elegans* is used extensively for laboratory studies interrogating mechanisms of aging and, increasingly, the potential efficacy of antiaging interventions. This species of roundworm is a favored platform for aging research due to the following reasons: 1. Its lifespan and signs of senescence are highly predictable. 2. There is a high degree of evolutionary conservation between *C. elegans* and mammals in putative aging pathways. 3. Factors such as exercise that are known to alter aging in humans and other mammals have similar effects in *C. elegans* [47]. Accordingly, increased longevity has been linked to mitochondrial fitness in both roundworms [57] and mammals [58]. Likewise, antiaging benefits of physical exercise have been linked to increased mitochondrial fusion in both *C. elegans* [47] and mice [46,59]. The relationship between mitochondrial dysfunction and aging in *C. elegans* has most commonly been interrogated through genetic manipulation of aging pathways. For example, mutational ablation of the *C. elegans* analog of mammalian mitofusins (Fzo-1) abbreviated worm lifespan, accelerated age-associated neuromuscular dysfunction and increased the prevalence of transgenerational mtDNA mutations [38,39]. These findings mechanistically link loss of mitofusin function to aging phenotypes in worms.

Here, we used recently described pharmacological tools to interrogate gain and loss of mitofusin functioning in *C. elegans* aging. Purists and experimental reductionists will assert that pharmacology is less precise than genetic manipulation to dissect physiological mechanisms of pathological states, and we do not disagree. But, as reviewed above, genetic manipulation as a phenotype-modifying strategy does not readily translate to the human condition. In contrast, mitofusin activators are under active investigation as potential therapeutics for age-related human neurodegenerative diseases [13,14,15,16]. Thus, our goal here was to test the notion that pharmacological mitofusin modulation using members of a drug class potentially headed for human use could alleviate aspects of aging related to mitochondrial dysfunction. The results of these proof-of-concept studies support this strategy, at least in nematodes.

Our experiments used complementary approaches to establish biological phenomena. Thus, mitofusin inhibition reduced worm lifespan while mitofusin activation prolonged survival; both of these experiments support a direct relationship between mitofusin activity and longevity. Moreover, we independently observed the life-prolonging effects of Chimera and 8015-P2, which have markedly different chemical structures but share the same effects on mitofusin activity and the same allosteric mechanism of activation [16,25,44]. 8015-P2 provided a greater survival benefit than Chimera, consistent with its being approximately ten times more potent as a mitofusin activator [16,25]. Finally, we observed synergy between pharmacological and genetic enhancement of mitochondrial fusion (i.e., a mitofusin activator plus DRP-1 gene ablation), suggesting that the mechanism by which mitochondrial fusion is increased is less important than the event. On the other hand, the loss of some of the longevity effect of mitofusin activation at the highest dose of the drug when combined with DRP-1 ablation suggests that a therapeutic window does exist for this approach.

Extended survival is not synonymous with prolonged youth. A youthful phenotype combines prolonged life with enhanced fitness at an age where it would normally be declining. Here, prior to any mortality (DOA 10), mitofusin activation increased physical vigor, which was manifested as the swimming rate, and enhanced tolerance to both thermal and oxidative stress. Importantly, mitofusin activation had no measurable effects on basal ROS levels in DOA 10 *C. elegans*, suggesting that healthspan benefits observed at this age had more to do with enhancing mitochondrial fitness than to preventing mitotoxicity.

## 5. Conclusions

These studies demonstrate that pharmacological mitofusin activation can both extend and enhance the quality of life in *C. elegans*. Activating mitofusins increases mitochondrial fusion-mediated repair. Although mitofusins have been considered an attractive drug target [41], pharmaceutically acceptable mitofusin activators have only recently been described. Preclinical studies have validated the potential for multiple chemical classes of mitofusin activators to interrupt or alleviate neurodegenerative diseases such as CMT2A and amyotrophic lateral sclerosis that, in human patients, are age-related [60]. Now that the therapeutic potential of this class of compounds has been established in worms, it will be interesting to determine how these findings may translate to mammalian aging.

## Data Availability

The raw data supporting the conclusions of this article are available, indexed to figure panel, in the online Supplementary data xlsx spreadsheet.

## Supplementary Materials

The following supporting information can be downloaded at https://www.mdpi.com/article/10.3390/cells15191789/s1, Original data: Supplementary data.xlsx.
